# Controlled-Release Carbamazepine Matrix Granules and Tablets Comprising Lipophilic and Hydrophilic Components

**DOI:** 10.1080/10717540802518157

**Published:** 2009-01-19

**Authors:** Nahla S. Barakat, Ibrahim M. Elbagory, Alanood S. Almurshedi

**Affiliations:** Department of Pharmaceutics, College of Pharmacy, King Saud University, Riyadh, Saudi Arabia

**Keywords:** Carbamazepine, Wet granulation, Compritol® 888 ATO, Hydroxypropyl methylcellulose, Dissolution studies, Swelling studies

## Abstract

The objective of this study was to investigate the effect of lipophilic (Compritol® 888 ATO) and hydrophilic components (combination of HPMC and Avicel) on the release of carbamazepine from granules and corresponding tablet. Wet granulation followed by compression was employed for preparation of granules and tablets. The matrix swelling behavior was investigated. The dissolution profiles of each formulation were compared to those of Tegretol® CR tablets and the mean dissolution time (MDT), dissolution efficiency (DE%), and similarity factor (f2 factor) were calculated. It was found that increase in the concentration of HPMC results in reduction in the release rate from granules and achievement of zero-order is difficult from the granules. The amount of HPMC plays a dominant role for the drug release. The release mechanism of CBZ from matrix tablet formulations follows non-Fickian diffusion shifting to Case II by the increase of HPMC content, indicating significant contribution of erosion. Increasing in drug loading resulted in acceleration of the drug release and in anomalous controlled-release mechanism due to delayed hydration of the tablets. These results suggest that wet granulation followed by compression could be a suitable method to formulate sustained release CBZ tablets.

The development of oral controlled-release dosage forms has attracted much attention in recent years. Hydrogels are being increasingly investigated for controlled-release (Kudela 1987). In addition the hydrogels have the ability to release the entrapped drug in aqueous medium and to regulate the release by controlling the swelling (Heller et al. 1983; Graham and McNeill 1984). Hydrogels can be applied for the release of both hydrophilic and hydrophobic drugs and charged solutes. Hydrogel provide the basis for implantation, transdermal and oral controlled-release systems. Hydrophilic polymers, in particular cellulose derivatives, have been widely used in the formulation of hydrogel matrices which satisfy the key criteria for the development of controlled-release oral solid dosage forms. The hydration rate of these polymers depends on the nature of substitutes and the degree of substitution. Once the polymer hydrates quickly enough to form a gelatinous layer, a change in polymer viscosity will directly change the dissolution rate. Usually two main mechanisms are involved, diffusion and erosion. In the case of cellulose polymer-based matrix, drug release can be described as being controlled by the rate of swelling (Akbari et al. 2000; Peppas and Simmons 2004). However, drug release in general is not purely swelling controlled, since it occurs mostly as the result of a combination of polymer relaxation and Fickian diffusion (Lowman and Peppas 2000). In practice for the controlling and programming of drug release from matrix devices, different types of modified cellulose polymers are usually employed, either alone or in mixtures with other swellable polymers (Aïnaoui and Vergnaud 2000) or with hydrophobic polymers (Lotfipour et al. 2004) which may alter the release mechanism and rate. More recently, hydrophobic polymers, Glyceride such as Compritol (glyceryl behenate) have been used for the preparation of controlled release formulations since they possess some very interesting characteristics, i.e. chemical inertness against other materials and excellent flow properties. Several studies have been made on the in vitro release from matrices comprising hydrophobic and hydrophilic components (Malamataris and Ganderton 1991; Kiortsis et al. 2005). Lipids may be suitable in this way as release modifiers for incorporation into cellulose matrices. The purpose of this study was to examine how diffusion and erosion combine in a matrix comprising an insoluble hydrophobic and hydrophilic gel-forming element using Compritol® 888 ATO and cellulose polymer (HPMC and Avicel) together with carbamazepine (CBZ) and employing a conventional wet granulation technique. The objectives of this work are: (i) to evaluate the physical characteristics of the prepared granules and matrix tablets; and (ii) to elucidate the effect of CBZ loading and of Compritol® 888 ATO:HPMC:Avicel® weight ratio on the release kinetics of CBZ from granules and matrix-tablets. A formulation without HPMC was also employed for comparison.

## Materials and methods

Carbamazepine was kindly supplied by Novartis Pharma (Cairo, Egypt). The powdered excipients were: Compritol® 888 ATO (Gattefossê, Saint Priest, France) used as insoluble hydrophobic (non-wetting) matrix component, Hydroxypropyl methylcellulose (HPMC, Methocel K15 M, DOW Chemicals and Colorcon, Orpington, UK) and microcrystalline cellulose (Avicel® PH-102; FMC Corporation, Hamburg, Germany) used as hydroplilic matrix-components. Other reagents and solvents employed were of analytical grade.

### Preparation and evaluation of granules

All powdered ingredients were passed through a 250 μm sieve before use for deagglomeration. Fifty gram batches of powder mixtures composed of CBZ, Compritol®, HPMC, and Avicel in contact drug:matrix forming excipient ratio 1:2 were tumble mixed for 20 min. The proportions of the matrix forming excipient (Compritol®:HPMC:Avicel) were 7:2:1; 6:3:1; 5:4:1; 4:5:1; 2:7:1; and 1:8:1 (given in Table 1). Ethanolic solution of 10% PVP was added at a slow steady rate to the blended mixtures. The quantity of alcoholic solution had been previously determined on the basis of over-wetting tests. The wet mass was allowed to pass through No 14 sieve. The passing granules were dried in an oven, at 40°C for 6hr to a moisture level of about 1% w/w, then left to cool down at room temperature. The 500–710 μm sieve fraction was obtained and stored in glass jars. Granules without any drug were also prepared to study the erosion and water uptake behavior of the inert matrix. The granules were evaluated on the basis of CBZ content, angle of repose, bulk (BD), and tap (TD) density. Also, the Carr's index was calculated by using the following equation:

(1) CI=TD − BD × 100/TD

**Table 1:** Composition of CBZ wet granulations comprising lipophilic–hydrophilic matrix components.

		Components			
		
Formula code$	Matrix component's ratio	CBZ*	Compritol®	HPMC**	Avicel® PH-102
A1	7:2:1	200	280	80	40
A2	6:3:1	200	240	120	40
A3	4:5:1	200	160	200	40
A4	2:7:1	200	80	280	40
A5	1:8:1	200	40	320	40
Control	9:0:1	200	360	—	40

$ All the formulations prepared into granules and tablet.

* CBZ:matrix ratio was kept constant at 1:2.

** Hydroxypropyl methylcellulose K15M.

### Preparation of matrix tablets

An appropriate quantity of dried granules (size fraction 710–500 μm) from each formulation (Table 1); enough to make 25 tablets, was weighed and placed in a glass container. Magnesium stearate 1% w/w was added and tumbled mixed for 5 min. Accurately weighed portions of lubricated granules from each formula containing CBZ equivalent to 200 mg were fed manually to the die of a single punch tableting machine equipped with flat faced punch of 9-mm diameter and compressed at the maximum force. The properties of the matrix tablets, such as CBZ content, friability, weight variation, thickness, and diametral tensile strength were determined. Control tablets containing Compritol®:Avicel® at 9:1 weight ratio were prepared under identical conditions.

### CBZ content of the granules and tablets

Fifty milligrams of granules or tablets were further ground into fine powder and suspended in 50ml acetonitrile in order to extract the CBZ content. The suspension was kept in an ultrasonic bath for 15min and then was centrifuged for 15min at 4000rpm and filtered through a 0.5μm. After suitable dilution of the supernatant the content of CBZ was determined by applying UV spectroscopy. Each determination was performed with two powdered samples.

### Differential scanning calorimeter (DSC)

About 2–5mg either pure drug, pure excipient, or drug:excipient physical and granulated mixture was analyzed in a Perkin-Elmer differential scanning calorimeter (Perkin Elmer DSC-7, Norwalk, CT), at a heating rate of 10°C/min, from 25 to 200°C. The samples were heated in sealed aluminum pans, under a nitrogen flow (20ml/min) and an empty sealed pan was used as reference. The apparatus was calibrated with indium (99.98%, m.p. 156.65°C).

### Fourier transform infrared spectroscopy (FT-IR)

The infrared spectra of the CBZ, Compritol®, HPMC, Avicel®, the physical mixture, and the prepared granules were obtained on a Fourier transform infrared spectrometer (Perkin-Elmer, Norwalk, CT) in order to detect the existence of interactions between CBZ and hydrophobic or hydrophilic excipients in the granulation. The samples were first ground gently in a mortar and mixed with KBr before being compressed into tablets. Scans were obtained at a resolution of 2cm^−1^, over a frequency range of 4000 to 400 cm^−1^.

### In Vitro release studies

The in vitro drug release was evaluated by using the USP/NF dissolution apparatus II (Erweka Apparatus, Germany). An accurately weight amount of granules, equivalent to 200 mg CBZ, or one tablet, was added to 900 ml of 1% sodium lauryl sulfate aqueous solution maintained at 37 ± 0.5°C. Rotational speed of the paddles was 75 rpm. Aliquots of 5 ml of dissolution medium were withdrawn at 15, 30, 60, and 120 min and then at regular intervals of 1 hr for up to7 hr, and replaced with equal volumes of fresh dissolution medium. The CBZ content was determined using a UV spectrophotometer (Ultrospec 2100 Spectrophotometer, UK) at 285 nm. Granules withouth CBZ were used as blank and their absorbance due to the lipophilic and hydrophilic excipients was negligible compared with that of the drug. The results of three determinations were expressed as CBZ % released.

### Water uptake (Swelling) of compacted matrix components

Swelling was evaluated as water uptake determined gravimetrically (Sutananta et al. 1995a, b). Compacts of the same size and shape as the matrix-tablets used for drug release testing were prepared without drug or magnesium stearate. They were placed in small baskets and soaked in vessels containing 100 mL of distilled water at 37 ± 1°C. At 0.25, 0.5 hr, and then at hourly intervals up to 7 hr, the previously weighed baskets containing the compacts were removed, gently wiped with a tissue in order to remove surface water, reweighed, and then placed back into the vessel as quickly as possible. The mean weights were determined for three compacts of each formulation, and the percentage of swelling (S%) was calculated according to the following relationship (Efentakis et al. 1997):

(2) $$\text{S}\%\text{=}\frac{{\text{W}}_{\text{s}}\;-\;{\text{W}}_{\text{d}}}{{\text{W}}_{\text{d}}}\times 100$$

where W_d_ and W_s_ are the dry and swollen compact weights, respectively, at immersion time *t* in the test liquid.

### Elucidation of release mechanism

Mechanism of CBZ release was elucidated by fitting zero order, first order, and Higuchi's square root of time equations (models) to the release data. *Qt* vs *t* for the zero order kinetic model; log (*Q*_0_ − *Q*_*t*_) vs *t* for the first order kinetic model; and *Q*_*t*_ vs √*t* for the Higuchi's model, where *Q*_*t*_ is the percentage of drug released at time *t* and *Q*_0_ is the initial amount of drug. The release constants (*k*_i_) and the correlation coefficient (*r*) were calculated by means of a computer EXCEL program.

Furthermore, to the CBZ release data of the granulations were fitted the simple power law Korsmeyer et al. (1983) expression which can best describe the kinetic of drug release from controlled-release matrices.

(3) $$Q_{t}/Q_{\infty }=kt^{n}$$

where *Q*_*t*_/*Q*_∞_ is the fraction of drug release at time *t*, *k* is the release rate constant and *n* is the release exponent that characterizes the mechanism of drug release. Values of *n* near 0.5 indicate predominantly diffusion control and of 1.0 correspond to zero-order release. To the CBZ release data of matrix-tablets were fitted the Peppas and Sahlin (1989) equation considering the two controlling mechanisms (Fickian and relaxational diffusion) of drug release from swellable matrices as additive:

(4) $$M_{t}/M_{\infty }=k_{1}t^{m}+k_{2}t^{2m}$$

where *M*_*t*_/*M*_∞_ is the fraction of drug released and the first term of the right-hand is the Fickian release contribution and the second term is the Case II relaxational release contribution. The coefficient *m* is the purely Fickian diffusion exponent and *k*_1_ and *k*_2_ are the kinetic constants.

To further characterize the drug release process, the mean dissolution time (MDT), the dissolution efficiency (%DE) and the similarity (*f*_2_) and difference (*f*_1_) factor of dissolution profiles between the commercial product (Tegretol® CR) and experimental formulations were calculated according to the following equations:

(5) MDT=∑j=1nt^jΔQ1∑j=1nΔQj

where *j* is the sample number, *n* the number of time increments considered, *t*^_*j*_ is the time at midpoint between *t*_*j*_ and *t*_*j*_ − 1, and Δ*Qj* the additional amount of drug dissolved in the period of time *tj* and *t*_*j*_ − 1 (Voegele et al. 1983).

(6) $$\%\text{DE}=\int_{\text{0}}^{t}\frac{y\times dt}{y_{100}\times t}\times 100$$

where *y* is the drug percent dissolved at time *t* and DE is defined as the area under the dissolution curve up to a certain time, *t*, expressed as a percentage of the area of the rectangle described by 100% dissolution in the same time (Khan 1975).

(7) $$f_{2}=50\times \log\left\{{\left[1+\left(1/\text{n}\right)\sum_{\text{j}=1}^{\text{n}}{\left({\text{R}}_{\text{j}}-{\text{T}}_{\text{j}}\right)}^{2}\right]}^{-0.5}\times 100\right\}$$

(8) $$f_{1}=\frac{\sum_{j=1}^{\text{n}}{\text{R}}_{j}-{\text{T}}_{j}\times 100}{\sum_{j=1}^{\text{n}}{\text{R}}_{j}}$$

where *n* is the sampling number, *Rj* and *Tj* are the percent dissolved of the reference and test products at each time point *j* (Moore and Flanner 1996). The similarity factor *f*_2_ is used to compare the difference and the difference factor (*f*_1_) measures the percent error between two curves over all time points. *f*_2_ value greater than 50 (50–100) represents equivalence of the two curves and the percent error is zero when the test and drug reference profiles are identical and increase proportionally with the dissimilarity between the two dissolution profiles.

### Statistical analysis

All the results were expressed as mean value and standard deviation (SD). In order to assess the statistical significance between the data, a single-factor analysis of variance (ANOVA) was carried out, using a computer program PC-INSTAT at a 5% significance level.

## Results and discussion

### Physical properties of the granules

Flowability of the granules was evaluated by determining the angle of repose and Carr index, CI, because it is a prerequisite to obtain solid dosage form with an acceptable weight variation. According to the literature data excellent flow properties are seen for granules with a compressibility index, CI, between 15–25 (Wells 1997). The compressibility index of the different granulations ranged between 14.3 and 26.1 and therefore indicate their suitability for tableting. Also the granulations showed acceptable angle of repose ranged between 28° and 35.5°.

### Evaluation of the tablet properties

All the granules comprising lipophilic–hydrophilic components were successfully compressed into tablets. It is noticeable that the drug content of all the tested tablets was found to lie between 96.49% and 100.55% of the labelled amounts that reflect good drug distribution and homogeneity. The diametral tensile strength was considered acceptable; it varied from 6.65 to 8.3 kg/cm^2^. Friability of all formulations was less than 1%.

### Differential scanning calorimetric studies

**Figure 1:**
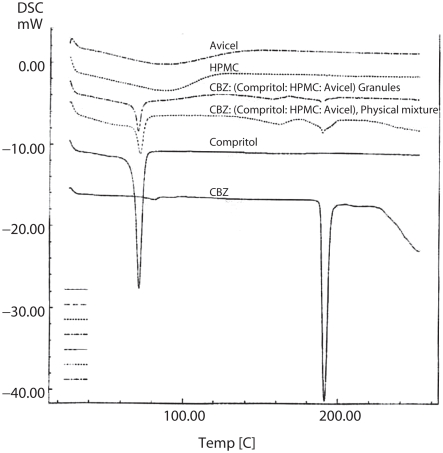
DSC thermograms of pure CBZ, Compritol®, HPMC, and Avicel® and of physical and granulated mixture at 7:2:1 Compritol®:HPMC:Avicel® weight ratio.

The differential scanning calorimeter curve of CBZ (Figure 1) displayed a single sharp endothermic peak at 198°C corresponding to its m.p. A single sharp peak at 72°C corresponded to the melting point of Compritol® and large shallow broad endothermic effects, over the temperature range 60–160°C, were observed for the polymers HPMC and Avicel, probably due to evaporation of adsorbed water. The DSC curve of the physical mixture of CBZ, Compritol®, HPMC, and Avicel® shows identical endothermic peaks to pure components but less intense due to the smaller concentration, indicating that the matrix forming components selected neither interfered with CBZ nor made any shift of its melting peak.

### FT-IR spectra

**Figure 2:**
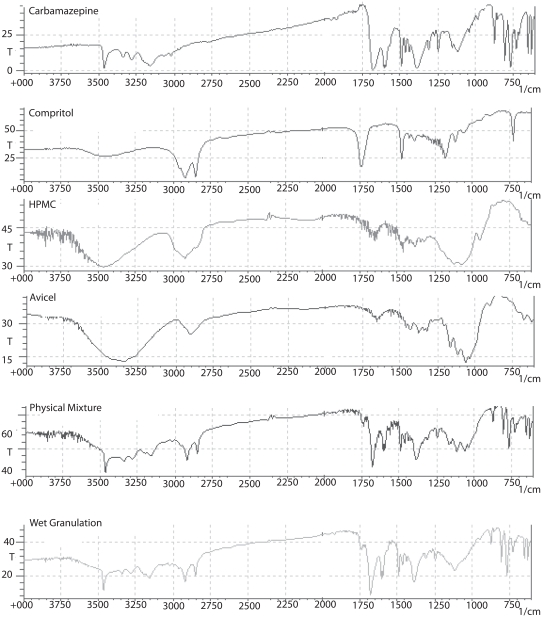
Infrared spectra of pure CBZ, Compritol®, HPMC, and Avicel® and of physical and granulated mixture at 7:2:1 Compritol®:HPMC:Avicel® weight ratio.

IR spectra of CBZ, Compritol, HPMC, Avicel, and their physical and granulated mixtures are shown in Figure 2. Bands of CBZ are observed at 3474 cm^−1^ (-NH valence vibration), 1686 cm^−1^ (-CO-R) vibration, 1603 and 1593 cm^−1^ (range of –C=C= and –C=O vibration and –NH deformation), and 1395 cm^−1,^ which are the same as described for CBZ polymorph II. The presence of –NH valence vibration at an intermediate wave number (3474 cm^−1^) was the major indicative sign that CBZ could be neither polymorph III (3464 cm^−1^) or polymorph I (3484 cm^−1^). The spectrum of wet granulations shows that the peak at 3474 cm^−1^ was partially reduced; as was expected, since CBZ content was only 40% w/w, but the main CBZ characteristic peaks were not affected.

### In vitro drug release kinetics from granulations

**Figure 3:**
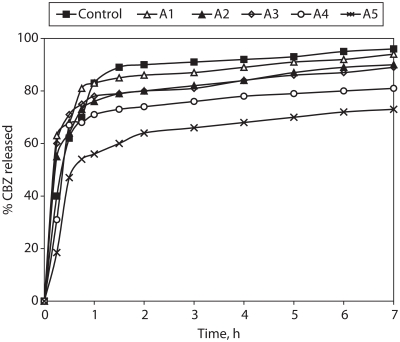
% Carbamazepine released from Compritol:HPMC matrix granules (size fraction 710–500 μm).

The dissolution profile of CBZ from the granules is shown in Figure 3. The release patterns showed fast dissolution and burst effect during the first hour. In the case of formulae with more than one weight ratio of Compritol®/HPMC or CBZ/HPMC (A1, A2, and A3), the drug release rate was not affected by the content of HPMC, since they produced a very similar release profile to the control formulation which did not contain HPMC. However, further increase of HPMC content (formulae A4 and A5, with less than one Compritol®/HPMC or CBZ/HPMC weight ratio) led to decrease of the burst effect and showed a more sustaining effect (less than 70% CBZ release over 1 hr). The release rate of CBZ considerably slowed after the first hour probably due to hydration of HPMC and formation of a gel layer with a longer diffusion path length as the content of HPMC was increased. This indicates that concentration of HPMC is an important factor which may control the mechanism and the rate of drug release (Alderman, 1984. Skoug et al. 1993; Wan et al.1993; Gao et al. 1996; Rekhi et al. 1999). Burst release is often observed prior to or during development of a diffusion barrier capable of controlling the penetration of dissolution medium and drug diffusion (Huang and Brazel 2001). Additionally when polymer concentration is low, the hydrated matrix would be highly porous with a low degree of tortuosity leading to low gel strength and rapid diffusion of the drug from matrix (Khurahashi, Kami, and Sunada 1996).

Table 2 summarizes the results of CBZ release modelling for the granulations under investigation. For the Korsmeyer et al. model results are given only with formula A4 showing less than 70% of drug release during the first hour. The goodness of fit for the various models ranked in the order: Higuchi ≅ Korsmeyer et al. > first-order > zero-order. The facts that drug release from granules follows the Higuchi and Korsmeyer et al. models and the values of the exponent *n* are around 0.5 in Table 2 (0.342–0.447) are indicative of diffusion controlled release.

**Table 2:** Fitting of release kinetic models to CBZ release data for wet granulations (size fraction 710–500 μm).

	Formula code							
	
Release model		A1	A2	A3	A4	A5	control^c^	Tegretol®
Zero-order	r**	0.765	0.938	0.767	0.842	0.713	0.665	0.939
	*k*_0_	6.531	7.493	8.482	8.472	45.590	5.272	11.880
First-order	*r*	0.856	0.989	0.843	0.923	0.848	**0.889**	0.995
	*k*_1_	0.146	0.112	0.163	0.144	1.728	0.326	0.427
Higuchi diffusion	*r*	**0.860**	**0.992**	**0.859**	**0.949**	**0.855**	0.815	**0.998**
	*k*_H_	23.69	22.55	24.63	25.72	32.67	19.24	36.6
Korsmeyer-Peppas*n*#	*r*	—	—	—	0.905	0.845	—	0.999
	*k*^−n^	—	—	—	80.31	47.83	—	44.46
		—	—	—	0.447	0.342	—	0.433

* Analyzed by the regression coefficient method.

** Correlation coefficient.

# Release exponent evaluated for < 70% released drug.

c Matrix composed of Compritol®:Avicel (9:1). Best fit in bold.

— Too rapid release to allow calculation for < 70% release.

Regarding the other dissolution indices, the Dissolution efficiency was relatively high (%DE7h 63.3–85.4%) and the change in MDT-80% was minimal (0.6–1.0 hr) for all the formulations under investigation, *p* > 0.05. The difference factors *f*_1_, presented in Table 3, reveal that all the prepared formulations were significantly different to the Tegretol®. Formula A5 (Compritol®:HPMC:Avicel® at 1:8:1 weight ratio) had a *f*_1_ value of 4.3, indicating that it has the closest dissolution profile to the reference (Tegretol®).

**Table 3:** Mean dissolution time (MDT), dissolution efficiency (%DE) and difference factor (*f*_1_) of release behavior between experimental wet granulations and matrix tablets and reference CBZ.

	Granules			Tablet		
		
Formula code	MDT# (h)	%DE*	*f*_1_**	MDT(h)	%DE	*f*_1_
A1	0.6	85.4	34.9	3.10	7.4	89.8
A2	0.8	80.7	26.4	3.34	13.8	81.9
A3	0.63	79.6	26.9	2.70	26.3	65.9
A4	0.6	72.5	13.1	2.60	43.5	44.8
A5	1.0	63.3	4.3	2.44	50.1	34.7
Tegretol				1.91	71.32	

# Mean dissolution time (MDT-80%) calculated from equation (5).

* Dissolution efficiency over 7 hr calculated according to equation (6).

** Difference factor calculated according to equation (7).

### In vitro drug release kinetics from matrix-tablets

**Figure 4:**
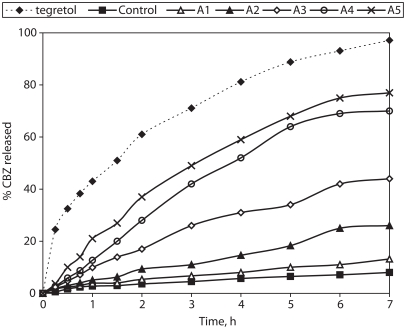
% Carbamazepine released from prepared matrix tablets and commercial product Tegretol®.

The release profiles obtained from the matrix-tablets and the Tegretol® tablets are presented in Figure 4. They show that the release rate from the control tablets (with Compritol®:Avicel® at a 9:1 weight ratio) was very slow. Also they show that the increase of HPMC content affects significantly the matrix tablet release behavior. The percentage of CBZ released over 7hr from the formulation of highest HPMC content (formula A5 with Compritol®/HPMC weight ratio 1:8) was 76.8%. In general the faster CBZ release rate with the HPMC increased content could be due to more rapid penetration of water into the matrix and/or more matrix erosion. However, a gradual disintegration of the swollen HPMC-based tablets was observed during the release studies. This may be explained by an axial expansion of the tablets as described by Rajabi-Siahboomi et al. (1994). Close examination of the HPMC containing matrix-tablets showed that the extent of their deformation was greater for those of higher HPMC content.

To analyze the mechanism of drug release from the matrix-tablets, the dissolution data were fitted to various kinetic models, the release kinetic parameters and the fitting ability (correlation coefficient, *r*) are listed in Table 4. The formulae A1–A3 give *n* values in the range of 0.624 to 0.884, corresponding to an anomalous diffusion mechanism. Also, both Higuchi model (Fickian) and first order kinetics were fitted similarly well. Increase of the HPMC content in the matrix-tablets (formulae A4 and A5) results in exponents *n* values (*n* = 1.01 and 0.938) which markedly exceed the value of 0.50 corresponding to diffusion controlled release and furthermore together with the good fitting of the zero-order model indicate significant contribution of erosion. Furthermore, the higher value of the relaxation constant, *kr*, compared to the diffusion constant, *kd*, in the Peppas-Sahlin model (equation 3), combined with the low CBZ solubility, indicate the prevalence of the erosion vs swelling mechanism. The MDT results showed almost insignificant difference due to increase of the HPMC content while the dissolution efficiency (%DE) result showed significant difference (Table 3). The difference factor *f*_1_ between the dissolution behavior of the experimental formulae and the reference (Tegretol®) are above 15, indirectly indicating significant differences between the experimental matrix-tablets as well.

**Table 4:** Fitting of release kinetic models to Tetretol release data for matrix-tablets.

	Zero Order		First Order		Higuchi model		Peppas-Sahlin model			
					
Formula code	*r*	*k*_0_(%h^−1^)	*r*	k_1_ (h^−1^)	*r*	k_H_ (%h^−1/2^)	*r*	*k*_d_ (%h^−m^)	*k*_r_ (%h^−2m^)	*n*#
A1	0.991	1.63	**0.997**	0.02	0.989	5.19	0.979	4.78	1.63	0.624
A2	**0.995**	3.72	0.992	0.04	0.976	11.7	0.997	8.76	2.91	0.846
A3	**0.997**	6.26	0.994	0.08	0.996	20.3	0.996	7.84	11.87	0.884
A4	**0.998**	10.9	0.992	0.19	0.992	35.4	0.999	7.71	27.72	1.010
A5	**0.996**	11.18	0.994	0.22	0.988	36.6	0.987	7.83	28.71	0.938
Experimental control C	0.664	5.272	**0.889**	0.33	0.815	19.24				0.668
Tegretol®	0.9393	11.88	0.995	0.427	**0.998**	36.6				0.433

* Analyzed by the regression coefficient method.

# Release exponent evaluated for < 70% released.

C Matrix composed of Compritol® 888ATO:Avicel (9:1). *k*_d_ and *k*_r_ calculated according to equation (4). Best fit in bold.

### Water uptake (Swelling) of compacted matrix components

**Figure 5:**
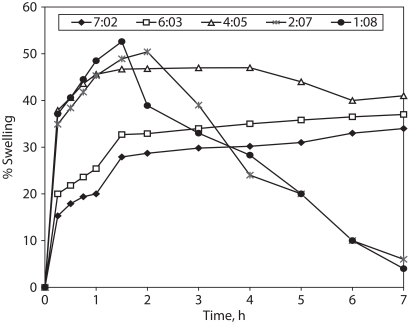
Water uptake (swelling %) of compacted matrix-forming components.

Figure 5 summarizes the results obtained from the hydration process of the compacted matrix components (tablets without CBZ). They support the dissolution results (Figure 4). The compacted matrix components of formulae A1, A2, and A3 (with more than one weight ratio of Compritol®/HPMC) exhibited relatively faster water uptake (swelling) during the first 1 hr of immersion followed by a steady hydration rate (water uptake plateau) for the next 6 hr. In contrast, the compacted matrix components of formulae A4 and A5 (with less than one weight ratio of Compritol®/HPMC) showed significant erosion which was becoming faster with the increase in the HPMC content. From the above mentioned we can conclude that the overall CBZ dissolution rate and, ultimately, availability for absorption should be controlled by the rate of matrix swelling, drug diffusion through the gel layer, and erosion of the outer gel layer (Roy and Rohera 2002).

### Effect of drug loading

**Figure 6:**
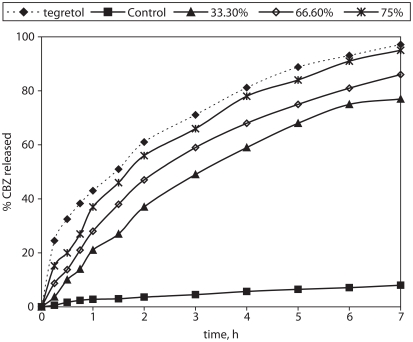
% Carbamazepine released from matrix tablets of increased CBZ loading.

The increase of drug loading from 33.3 to 75% resulted in acceleration of the release rate (Figure 6), which is attributed to increased presence of drug particles close to the surface of the matrix-tablets. Furthermore, the kinetic model fitting results show that increase of CBZ loading from 33.3% to 75%w/w causes a significant decrease in the release exponent *n* (from 0.939 to 0.637), which means shift of erosion-controlled (zero-order) release to anomalous mechanism. This may be caused by a delayed hydration of the matrix-tablets because of the poor water solubility and the hydrophobicity of the incorporated CBZ. Polymer erosion is less evident for tablets of high CBZ loading (75% w/w), and this was reflected on higher value of diffusion constant *kd* (26.753 %h^−m^) in comparison of relaxation constant, *kr* (8.651%h^−2m^) in the Peppas-Sahlin equation. On the contrary, Zuleger and Lippold found that for acetophenetidin the release was faster for the tablets with higher drug loading and this caused a significant increase in the release exponent at values strongly exceeding the expected *n* values for erosion controlled, zero order release. It was attributed to increased release area due to erosion and disintegration of the tablets.

## Conclusions

Combination of Compritol® with HPMC and Avicel as matrix former offers a flexible system able to sustain the CBZ release (85% release after 7 hr). Since the hydration ability and the mechanical strength of the gel developed in combination with the mechanical stress applied in the stomach and intestine can influence the integrity and subsequently the in vivo drug release mechanism, the formulation containing 75% w/w CBZ in a matrix composed of Compritol®:HPMC:Avicel at 1:8:1 weight ratio was selected for further in vivo study in dogs (Barakat et al. 2008).
